# Early childhood caries: Exploring the ethical implications for dental neglect in South Africa

**DOI:** 10.4102/hsag.v30i0.3129

**Published:** 2025-11-06

**Authors:** Nashna Rampersad, Nadia Mohamed

**Affiliations:** 1 Department of Community Dentistry, Faculty of Dentistry, University of the Western Cape, Bellville, South Africa; 2 Department of Paediatric Dentistry, Faculty of Dentistry, University of the Western Cape, Bellville, South Africa

**Keywords:** dental neglect, child oral health, early childhood caries, oral health advocacy

## Abstract

**Background:**

Dental neglect is defined as the ‘wilful’ failure of a parent and/or guardian to provide optimal oral healthcare, leading to pain and infection. Child abuse and neglect, including dental neglect, are significant public health issues with long-term consequences for victims.

**Aim:**

Given the early childhood caries (ECC) prevalence and socio-economic circumstances of South Africa’s (SA) population, the aim of this review is to explore whether ECC constitutes child neglect in SA.

**Method:**

This study explored existing literature on ECC prevalence in SA and its association with dental neglect. It examined SA’s child protection policies and considered the socio-economic context contributing to dental neglect.

**Results:**

Evidence suggests that dental neglect, including untreated caries, may indicate broader neglect. However, SA’s historical injustices, poverty, unemployment and lack of access to quality healthcare and education have resulted in high economic disparity among its population, perpetuating ill-health. Early childhood caries in SA is a broader social problem shaped by political, social and economic forces. Societal neglect shifts focus away from parents and towards examination of harmful government policies and actions which impose constraints on families, leading to diseases such as ECC.

**Conclusion:**

Child safety, optimum healthcare and prevention of neglect should be priorities of SA’s health and government systems as the country works towards universal health coverage and health system reform.

**Contribution:**

This study raises awareness about dental neglect as a child health issue, addresses the root causes of ECC within a societal context, advocates for policy changes and aligns with national and global health priorities.

## Introduction

Childhood abuse and neglect are significant public health issues because of the long-term consequences they have on victims (Shaw & De Jong 2018). As a vulnerable population, children are protected by South Africa’s (SA) Constitution and its *Childcare Act of 2005*. The Act focuses on formal measures to protect children from harmful actions and negligence, particularly by those directly responsible for their care (Burton et al. 2015). However, in SA, the rate of child maltreatment is extremely high. Schmidt and Azzi-Lessing (2019) suggested that this is partly because of SA’s extreme poverty and widespread violence.

In the context of child neglect, early childhood caries (ECC) represents a significant burden in SA. In 2017, the global incidence of oral health conditions was the third highest among all health problems (Kimmie-Dhansay et al. 2021). It is well recognised that ECC has notable long-term consequences. Peerbhay and Barrie (2012) reported that the Western Cape’s public health system was severely burdened with a relatively high number of children requiring dental treatment under general anaesthesia as a result of ECC. Dental extractions are the primary management sought because of the extent and severity of disease when presenting for clinical care (Peerbhay & Barrie 2012). The high prevalence of ECC and high treatment needs highlight a concern and raise the question of whether a diagnosis of ECC would constitute dental neglect. Considering that the purpose of public health is to promote and protect population health as well as to prevent or reduce morbidity and premature mortality (Mann et al. 1999), this discussion explores the ethical implications of ECC as a potential case of child (dental) neglect in SA.

The purpose of this article is to explore the ethical implications surrounding ECC as a potential case of dental neglect in SA. To achieve this, this discussion will examine the prevalence of ECC in SA and consider whether ECC can be explicitly viewed as a form of dental neglect within the South African context. In addition, the role of the South African public health system in preventing (child) dental neglect will be described. By addressing these key aspects, this discussion aims to shed light on the complex ethical considerations surrounding ECC and will contribute to a better understanding of its classification as neglect in SA.

### Early childhood caries in South Africa

Early childhood caries is defined as any decayed deciduous tooth surface (cavitated or non-cavitated), missing teeth (as a result of caries), or filled teeth in children under the age of 6 years (American Academy of Pediatric Dentistry 2020). Furthermore, severe early childhood caries (s-ECC) is any sign of smooth-surface caries in children younger than 3 years (particularly affecting the maxillary anterior teeth). Severe early childhood caries is a more severe form of decay that is commonly associated with poor infant feeding practices, i.e. nocturnal and prolonged bottle-feeding and breastfeeding (American Academy of Pediatric Dentistry 2020).

Approximately 514 million children globally suffer from dental caries of the primary dentition (World Health Organization [WHO] 2022). During the 10-year period between 2009 and 2019, among all the WHO regions, the African region was reported to record the highest (87.2%) increase in case numbers for dental caries of primary teeth in children between the ages of 1 and 9 years. Thus, the burden of dental caries in this region remains significantly high, affecting almost 374 million children in 2019 (WHO 2022).

**FIGURE 1:**
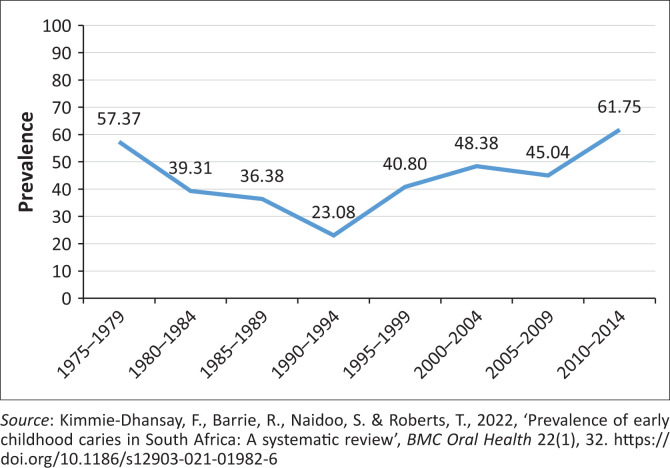
Early childhood caries prevalence by year: South Africa (1975–2014).

In SA, there is a significant burden of ECC (Smit, Barrie & Louw 2017). Although the prevalence of ECC in SA had previously decreased from 1975 to 1994, it has since increased from 1995 to date (Kimmie-Dhansay et al. 2022), as illustrated in Figure 1.

Previous studies have reported that South African children have a high caries experience, specifically with a high prevalence of ECC and substantial unmet dental treatment needs (Smit et al. 2017). Kimmie-Dhansay et al. (2022) further reported a lifetime ECC prevalence of 44.5% over a 40-year period among South African children. In low socio-economic areas surrounding the Tygerberg Oral Health Centre, Mohamed and Barnes (2018) reported an ECC prevalence of 71.6% in children between the ages of 6 months and 6 years. The study reported that 62.2% of the children were diagnosed with severe ECC. Active caries was present in 62.8% of children aged 2 years and 77.9% of 3-year-old children. This is a major cause for concern.

**FIGURE 2:**
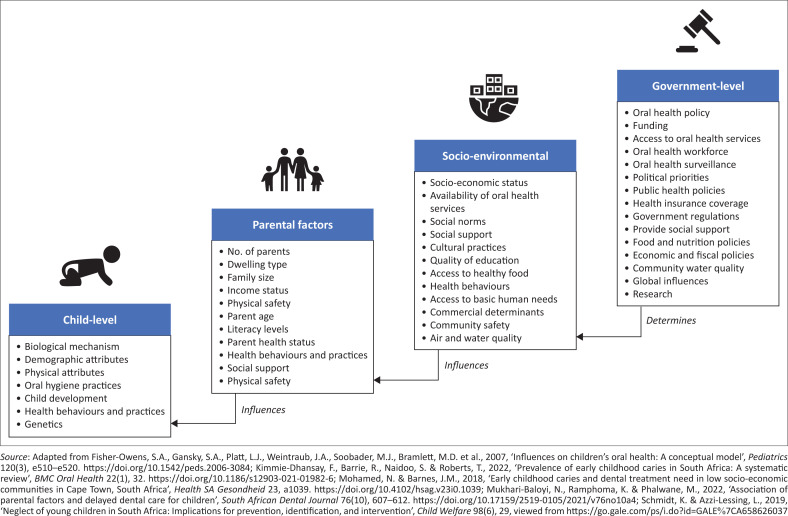
Stakeholders and factors impacting child oral health.

Children’s oral health is influenced by a range of stakeholders, including the child, parents, socio-environmental factors and government policies. Children are extremely reliant on others to meet their basic needs, making them particularly vulnerable. Furthermore, children are not in a position of autonomy and rely on decisions made for them by others, which increases their vulnerability (Bagattini 2019). Parents are responsible for the child’s basic needs, including diet, personal hygiene and access to healthcare. The stakeholders and factors that impact a child’s oral health are illustrated in Figure 2.

At child level, Figure 2 describes the aetiological factors that must all be present at the same time for the initiation and progression of the biological mechanisms of dental caries. These are fermentable carbohydrates (substrate), cariogenic microorganisms, a susceptible host (tooth surface) and time (Bradbury-Jones et al. 2013). Parents would therefore play a very important role in the prevention of dental disease in their children, influencing their child’s health behaviours and practices.

Additionally, research suggests that the aetiology of childhood caries is multifactorial and has evolved into a multilayered complex interaction between the general biological factors and socio-environmental factors (Hugar et al. 2016). The social determinants of disease or community-level influences that affect dental caries prevalence also need to be taken into consideration. These social-behavioural risk factors that contribute to dental caries in children, include the oral healthcare service system, risk behaviours (oral hygiene practices and sugar consumption), environmental factors (good quality drinking water, proper sewage system, proper hygiene practices and adequate nutritional status) as well as social-cultural factors (level of education, occupational and income status of the parent, race, lifestyle and social networks). Studies have reported that children in lower socio-economic living situations had higher incidences of dental caries (Sajadi et al. 2018).

Chronic malnutrition among children has been associated with increased caries rates (Seow 1998). However, several other social-behavioural factors play a role in the development of ECC specifically. Parental influences play a major role in the prevalence of dental caries among children. Sajadi et al. (2018) suggested that mothers with poor oral hygiene practices and frequent consumption of sugar-based snacks increased the likelihood of neonatal infection transfer (Sajadi et al. 2018). Furthermore, family living conditions play a significant role in ECC development, whereby changes in the family set-up (i.e. single-parent households) may affect the ability of parents to adequately prioritise and care for the oral health of the child, which increases the risk of dental caries in children (Sajadi et al. 2018). It is estimated that children from single-parent families had a 2.3 times higher incidence of ECC compared to children from two-parent families (Plutzer & Kierse 2010). Urbanisation and the increased access to refined sugars have also been reported as factors that have contributed to the increased prevalence of ECC (Sajadi et al. 2018). The rising prevalence of ECC in SA may be attributed to the process of urbanisation over the past 28 years since the end of SA’s apartheid era (Kimmie-Dhansay et al. 2022). By recognising the dynamic interplay of these factors, it becomes apparent that ECC poses a significant challenge to the underlying nutritional issues, which affect the well-being of South African children.

Among children of the Western Cape Province (SA), Smit et al. (2017) reported high unmet treatment needs, with the majority of carious lesions remaining untreated among these children. In a study assessing ECC and dental treatment needs of children of low socio-economic communities in Cape Town, Mohamed and Barnes (2018) reported that the majority (67.5%) of the children (> 6 months old to < 6 years old) in their study had evidence of dental caries that required some form of dental treatment. Using data from the previous National Children’s Oral Health Survey (1999–2002), it was determined that the unmet treatment need of 4–5-year-old children in the Western Cape was 93.3% in 2004, compared to 94.3% in 2018, which demonstrated that the issue has not been adequately addressed to date. This is a problem in other parts of SA as well (Mohamed & Barnes 2018).

**FIGURE 3:**
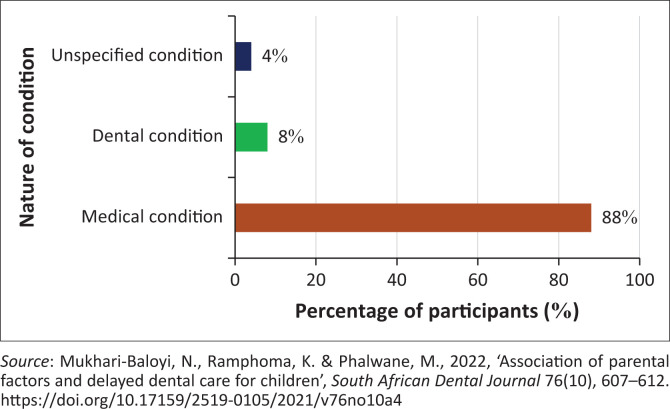
Circumstances that prompt parents to seek professional care for children.

A study investigating the parental factors associated with health-seeking behaviours, specifically seeking dental care for their children (< 6 years old) in the Tshwane district of SA, found that parental factors were significant risk factors for the development of ECC. Unsurprisingly, as illustrated in Figure 3, when assessing the association of parents’ knowledge and attitude about oral health and delayed dental visits because of pain, immediate healthcare was prioritised for acute medical conditions over general medical or dental conditions (odds ratio [OR] = 1.27). However, overall general medical conditions in children were not observed to take precedence over dental conditions, specifically for dental pain (OR = 0.85). Less than 10% of the parent study population reported that they would seek immediate dental care for their child’s dental conditions (Mukhari-Baloyi, Ramphoma & Phalwane 2022).

The majority of parents had negative perceptions of oral health. Almost half (47.4%) of the parent study population relied on home remedies and self-medication to treat their children’s dental pain, thereby delaying dental visits. Parents were 78% less likely to abandon home care and seek dental treatment when dental pain persisted, and they were 61% less likely to do so when the problems did not resolve (Mukhari-Baloyi et al. 2022). In addition, unemployment, a lack of education and poor oral health perceptions of parents were found to contribute to delays in dental consultations (Mukhari-Baloyi et al. 2022). Furthermore, less educated parents were more likely to delay or skip their children’s dental appointments (Mukhari-Baloyi et al. 2022). In impoverished settings, routine (preventive and curative) dental care was not prioritised (Mukhari-Baloyi et al. 2022). This was frequently viewed by parents as supplementary or optional treatment, with the exception of tooth extractions, which were viewed as necessary and definitive (Mukhari-Baloyi et al. 2022). Given the high prevalence of ECC and the unmet treatment needs among children in SA, the question arises as to whether a diagnosis of ECC would constitute dental neglect.

### Dental neglect

Dental neglect is defined as the ‘wilful failure’ of a parent (or guardian) to seek and complete treatment required to provide a level of oral health to enable optimal function as well as freedom from pain and infection (American Academy of Pediatric Dentistry 2020). Alternatively, the British Society of Paediatric Dentistry defined dental neglect as a ‘persistent failure’ rather than a ‘wilful failure’ to seek dental care (Harris & Whittington 2016).

Dental neglect is classified as either ‘active, passive, or self-neglect’, and its diagnosis is based on the root causes of dental neglect (Kiatipi et al. 2021). Active neglect occurs when parents or guardians purposefully fail to provide their child with the necessary treatment for optimal health and well-being (Kiatipi et al. 2021). On the contrary, passive neglect is when parents or guardians may be unaware of the oral health problem affecting their child and the consequences of not taking action or are unable to afford or obtain the appropriate oral healthcare that is required for their health and well-being (Kiatipi et al. 2021). As is the case in many low- and middle-income countries, additional limitations may include the availability of oral health services or challenges such as disabilities or financial difficulties. Lastly, self-neglect is defined as neglect caused by a child’s inability to express or meet his or her own needs because of physical, mental or developmental disabilities (Kiatipi et al. 2021).

Alternatively, dental neglect can also be classified as dental-prevention neglect and dental-treatment neglect. Dental-prevention neglect is the failure to implement basic oral health preventive measures, whereas dental-treatment neglect is the failure to provide appropriate professional dental care (Kiatipi et al. 2021). In both scenarios, there is the potential for untreated dental caries, traumatic dental injuries and dental pain or infection, thus affecting the quality of life of a child (Kiatipi et al. 2021).

Dental neglect, which is often associated with oral diseases, can significantly affect a child’s well-being and frequently results in a failure to seek dental care. Bradbury-Jones et al. (2013) further shared that dental neglect can be recognised in isolation or with other signs of neglect; more specifically, that untreated dental disease has become more widely recognised as a useful indicator of broader child neglect. Evidence suggests that children who have been abused or neglected have higher rates of tooth decay than the general population (Bradbury-Jones et al. 2013). Given this and the global prevalence of ECC, numerous studies suggest a correlation between ECC and child neglect (Bradbury-Jones et al. 2013; Scorca et al. 2013).

Dental neglect cannot be diagnosed solely on the basis of clinical evidence and the presence of dental caries. Various other dental and non-dental aspects need to be considered. As a result, no specific diagnostic criteria for dental neglect have been established, nor has a threshold number of carious teeth been determined that would constitute dental neglect. The question is whether this definition can be applied to the South African context.

Dental neglect is frequently the result of a combination of risk factors. These factors may include a child’s disability, parental ignorance (or depression), a lack of finances, domestic violence, as well as a lack of perceived importance of oral health (Scorca et al. 2013). Especially in SA’s context, several political, social and economic forces shape families’ living conditions and the range of opportunities and services available to them (Rampersad & Mohamed 2023; WHO 2022).

Under these circumstances, Asaka et al. (2020) reported that dental neglect, particularly in the form of untreated dental caries, was linked to socio-economic status and exhibited significant incidence and prevalence rates among population groups that were economically disadvantaged. In addition, not seeking treatment for a child with dental caries was more widespread among individuals with a low level of education, individuals who resided in rural regions, whose parents had a history of dental caries and who had poor dental hygiene practices (Fernández et al. 2020).

Asaka et al. (2020) further demonstrated that children with dental neglect were more frequently from less affluent families, even after adjusting for sex, grade and lifestyle factors of children and parents. Moreover, unhealthy lifestyles were also associated with a low socio-economic status. In addition, child poverty directly contributed to maltreatment, particularly neglect, as a result of parents’ decreasing ability to meet their children’s basic needs (Asaka et al. 2020).

### South Africa’s socio-economic situation

The South African population is unfortunately plagued with several challenges, which also serve as social determinants of health and, incidentally, share the same risk factors for ECC (Kimmie-Dhansay et al. 2021). Furthermore, the socio-economic status of the South African population is distinctly divided, with a high Gini coefficient (0.67). This indicates that high economic disparities exist among this population (Francis & Webster 2019) and could contribute to disparities in access to essential health and oral health services (Kimmie-Dhansay et al. 2021). Although access to basic healthcare has improved in SA over the past 20 years, profound oral health disparities continue to plague the population (Magan 2020). There are two distinct parallels that exist in the country. On the one hand, 20% of the country’s population are insured (Maphumulo & Bhengu 2019) and have optimal access to oral healthcare through the private sector, and on the other hand, the remaining 80% of the population are not insured (Maphumulo & Bhengu 2019) and rely heavily on the public health system to deliver accessible and equitable healthcare.

It has been well-established that poverty is a precursor to poor health. Poverty is a major contributing factor of illness and a barrier to receiving the necessary healthcare (The World Bank 2014). The poor cannot afford the costs of seeking healthcare (including transportation), purchase items necessary for good health, understand information on appropriate health-promoting practices or have the necessary voice to make social services work for them (The World Bank 2014). Similarly, in oral health, poverty is one of the greatest indicators of oral health disparities, which is followed closely by racial, ethnic and geographic indicators (Lalumandier & Molkentin 2004). In 2019, the rural population was reported to have made up 33.14% of SA’s population, with reports suggesting that almost half the country’s population was estimated to be living in poverty (StatsSA 2021) and that 35.4% of SA’s population was unemployed (StatsSA 2022b).

In 2020, 33 899 babies (3.8%) were born to mothers aged 17 years and younger, with the youngest mother being 10 years of age at the time of delivery. Furthermore, in the same year, more than 60% of the total births registered were recorded without paternal details (StatsSA 2021). A *General Household* survey conducted by *Statistics SA* revealed that subjectively poor households in SA are typically headed by a black African female who is younger than 35 years, who lives in a rural area in a rural-based province and has a low level of education. The house typically has between 0 and 1 bedroom, and the general health of its household head was reported to be poor. All economically active individuals (> 15 years of age) were more likely to be unemployed in these households, which were in the lower quintiles of the income distribution (StatsSA 2022a).

As a result, most of SA’s population is dependent on public health and social services to meet the demands of the population (Maphumulo & Bhengu 2019). Although the delivery of quality and equitable healthcare is a constitutional obligation in SA (Maphumulo & Bhengu 2019), many disadvantaged communities have limited access to healthcare, especially oral healthcare services. Ramphoma (2016) reported that the oral health disparities in SA continue to widen, especially in the disadvantaged and vulnerable groups, and are partly attributed to the unequal distribution of oral health services in SA. This is further exacerbated by the lack of oral health facilities and oral health workforce (Ramphoma 2016). Communities within these populations are predicted to experience the highest burden of oral diseases (Ramphoma 2016). Subsequently, many South Africans defer dental treatment, which could result in long-term effects on their physical and psychological well-being (Moodley & Singh 2018), thus placing South African children at an unfortunate and increased risk for developing ECC.

In the Western Cape Province of SA, Smit and Osman (2017) found that the basic oral healthcare package in public dental clinics in the province was not readily available and, further, disproportionately distributed among rural and urban public health facilities. In addition, although it is estimated that 80% of the South African population depends on public oral health services (Maphumulo & Bhengu 2019), Ramphoma (2016) reported that only one quarter of South African dentists were employed in the public health sector. This indicates that the existing oral health workforce in the public sector is too overburdened to address basic prevention needs for ECC and dental neglect.

These factors distinguish SA and its disease and risk profiles from other countries at comparable stages of development (Kimmie-Dhansay et al. 2021). Considering the socio-economic status of the country, the state of its public health system, the prevalence of ECC and the high levels of untreated dental caries among South African children, the issue extends beyond parental responsibility. Most parents do not ‘wilfully’ or ‘deliberately’ fail to seek oral healthcare for their children; rather, they are constrained by the inadequate and inequitably distributed public dental services. This neglect reflects broader systemic failings of the South African government, rooted in historical injustices, persistent poverty, high unemployment and limited access to quality healthcare and education, factors that perpetuate deep economic disparities and ill-health. Consequently, ECC emerges as a societal problem driven by political, social and economic forces, where harmful policies and insufficient service provision indirectly contribute to this persistently prevalent condition.

### Addressing areas of concern

The discipline of public health involves an obligation to care for the well-being of communities and possesses a measure of authority to carry out this obligation. At the core of public health ethics is the requirement to exercise power to ensure the health of populations while avoiding abuses of such power (Thomas et al. 2002). The ethical principle of utilitarianism, which seeks the greatest good for the greatest number of people, is a fundamental but often unstated assumption in public health (Mann et al. 1999). Human rights are concerned with promoting and protecting individuals’ well-being by ensuring respect for their rights and dignity. The ethical principle of autonomy is a central concern in human rights. Importantly, both human rights and public health have increasingly recognised the critical role of the societal environment in both health and the realisation of human rights (Mann et al. 1999). This is further emphasised by the American Public Health Association (2019), by suggesting that through obligatory public health support, society can be expected to ‘live flourishing lives’ with disabilities or illness. In this context, flourishing is less concerned with biological function and more concerned with the social conditions of capability and opportunity upon which health and numerous other health-related benefits depend (American Public Health Association 2019).

Notably, the opposite of human flourishing is not only disease or ill-health but also power and control, inequality, discrimination, exploitation, exclusion, distress and despair – in other words, the suppression and denial of optimal human self-actualisation and flourishing of society’s people (American Public Health Association 2019). The purpose of public health is to promote and protect population health, as well as to prevent or reduce morbidity and premature mortality. It comprises actions through the organised efforts of society and necessitates shifting the distribution of health risks through addressing the underlying social, environmental and economic conditions (Besnier et al. 2021). Therefore, when considering public health measures to protect vulnerable populations, such as children, these principles are essential. Consequently, both the South African public health system and numerous government industries have a strong reciprocal obligation to protect their children (Ramphoma et al. 2022) and, further, play a major role in addressing and preventing dental neglect among the country’s child population.

Scorca et al. (2013) suggested that to address this public health problem, it is essential to recognise protective factors against child neglect, such as parental recognition of a problem that prompted them to seek help and supportive grandparents or extended family members with a vested interest in the child’s well-being. Once parents are informed by a healthcare professional about their child’s condition, the specific treatment required and the processes involved for obtaining the necessary treatment, these parents are expected to comply. In Italy, if parents fail to comply, the case is required to be reported to child protective services, as the parents would be deemed to be negligent (Scorca et al. 2013).

However, in SA, the public health problem of ECC is part of a broader social problem, which cannot be addressed in isolation. Parental factors such as poverty, literacy level, unemployment and inequality continue to hinder access to dental care for children of poor families. Delays in the utilisation of routine preventative, promotive and curative services would therefore worsen oral health outcomes among the poor (Mukhari-Baloyi et al. 2022).

Importantly, Schmidt and Azzi-Lessing (2019) argued that SA has a degree of societal neglect as a result of the political, social and economic forces, which shape families’ living conditions and the extent to which opportunities, services and support are available. This concept of neglect shifts the focus away from parents or guardians and towards an examination of the policies and actions of governments, service providers and public utilities that harm children through the conditions and constraints imposed on families (Rampersad & Mohamed 2023). This is largely responsible for the prevalence of many diseases, such as ECC in SA (Schmidt & Azzi-Lessing 2019).

## Implications and recommendations

Although SA has comprehensive laws and policies related to child protection, children continue to receive fragmented services across all sectors, which offers poor support for their long-term well-being (Jamieson, Sambu & Mathews 2017). Therefore, addressing the issue of ECC within SA’s context requires a multifaceted approach, especially because of the high costs associated with oral healthcare and the known limitations in oral health service coverage by government programmes across many African countries (WHO 2022). Mitigating these challenges requires specific interventions at macro- and micro- levels.

At a macro level, improved oral health and integrated health promotion policies are required. Moreover, children need to be shielded from the impact of actions such as aggressive marketing of sugar-rich foods and related products. In addition to this, an actionable and well-resourced oral health policy needs to be endorsed in order to effect change.

Furthermore, acknowledging oral diseases, including ECC, as critical public health concerns on both global and regional scales will help sustain and amplify the efforts of all stakeholders involved. The 74th World Health Assembly, WHO (2021), adopted resolution WHA74.5 on oral health and emphasised integrating oral health into the non-communicable disease agenda and proposed the inclusion of essential oral health interventions in universal health coverage benefits. This resolution urged all Member States to transition from the traditional curative approach in oral healthcare towards more preventive and promotive approaches (WHO 2021). This recognition could facilitate the inclusion of an ECC-related indicator into SA’s District Health Information System, which may potentially ensure that ECC receives the necessary attention and resources it urgently requires to improve child health outcomes.

In addition to policy reform, health promotion initiatives must become more widely accessible to this child population. As much as oral health practitioners can be trained to recognise the signs of dental neglect, their efforts are often constrained by limited oral health service availability and a lack of innovative, context-specific disease prevention strategies. Consequently, practitioners depend heavily on strong governance and leadership within the oral health sector to enable meaningful impact. This complex challenge of ECC in SA is more than a health challenge; it reflects the deep-seated socio-economic inequalities in the country. The South African healthcare system is required to not merely focus on ‘corrective’ or ‘curative’ forms of care but to protect its children from the burden of ECC. Therefore, addressing this problem with a strategic and stepwise approach may assist with more contextual and sustainable outcomes.

## Conclusion

Untreated dental caries in children could be a sign of a broader neglect, alerting health providers to the need for the necessary assistance to prevent further harm or injury (Cukovic-Bagic et al. 2013). As the dental team is in a unique position to recognise signs of abuse and neglect (Mohamed & Naidoo 2014), *Section 110* of the *Children’s Act* mandates that dentists, among other key professionals, report any reasonable suspicion of child neglect and abuse.

It is, however, essential that ECC and child neglect in SA are considered contextually. ‘Child neglect’ implies a parent’s failure to meet their child’s basic needs is intentional or deliberate and has major legal ramifications if reported. This assumption may be correct in cases of selective neglect because of a child’s characteristics or when parents or caregivers have the capacity but fail to adequately supervise a child or provide emotional support and stimulation required for optimal development. In these instances, parents should be held liable, and government intervention becomes necessary. This is reflected in SA’s child protection policies, which require only ‘deliberate’ forms of neglect to be reported to the relevant authorities (Jamieson et al. 2017). Given SA’s rising rates of poverty and the strong correlation between family poverty and child neglect, it is likely that most situations in which parents are unable to provide adequate care for their children are caused by poverty rather than deliberate intent (Schmidt & Azzi-Lessing 2019). Thus, from a legal perspective, it may not be considered as child neglect. Furthermore, labelling this as child neglect has the undertone of ‘victim-blaming’, which is in actual fact more harmful.

The persistent inadequacy of SA’s public dental services in addressing its community’s oral health needs can be regarded as systemic neglect, which raises serious ethical concerns.

As SA endeavours towards *Universal Health Coverage* and prepares for a health system overhaul through the *National Health Insurance Bill*, child protection, care and the prevention of neglect are at the forefront (DICAG 2016). A more workable, context-specific oral health policy could provide the appropriate resources and realise the implementation of a proper prevention programme against ECC and, in some instances, prevent this passive form of child neglect in SA (Rampersad & Mohamed 2023). Additionally, raising awareness of the public health problem of ECC among other health professionals can facilitate early reporting of the disease. This will prevent severe disease progression and the accompanied long-term consequences of ECC.
